# The Man of Genius

**Published:** 1892-01-30

**Authors:** 


### EDTOR'S LETTER BOX.

[Our correspondents are reminded that prolixity of statement is a great bar to publication, and that brevity of style and conciseness of statement greatly facilitate early insertion.]

### "THE MAN OF GENIUS."

CYRIL BENNETT writes: It is a little unfortunate for Lombroso's theory that one of the strongest and sanest men that ever lived—William the Silent—should happen to have been a man of stupendous genius; not only a great patriot, ruler, soldier, courtier, and diplomatist, but a writer of the highest order, whose powerful letters and despatches, in various languages, fill many large volumes; and that this personification of sanity should also have been the father of strong children, amongst them being more than one strong and sane man of genius. It seems that if a person in any age or country has, justly or unjustly, made a name in letters or art, and if, at any period of his life, he has suffered from headaches, bilious attacks, indigestion, sensations of giddiness, or moral perversion, the professor regards him as a man of genius, and as a congenitally neurotic person, though he may well be neither one nor the other, and then draws the conclusion that genius is a diseased condition. It is an undoubted fact that most healthy persons suffer, at some time or other, from one or more of the above-named ills; and surely no one is worthy of the name of genius who is not highly endowed in all directions, with surplus energy at his disposal, with which to excel in some special direction, not necessarily in letters or art.

